# Recognizing the value of meta-research and making it easier to find

**DOI:** 10.5195/jmla.2023.1758

**Published:** 2023-10-02

**Authors:** Elizabeth R. Stevens, Gregory Laynor

**Affiliations:** 1 elizabeth.stevens@nyulangone.org, Department of Population Health, NYU Grossman School of Medicine, New York, NY.; 2 gregory.laynor@nyulangone.org, NYU Health Sciences Library, NYU Grossman School of Medicine, New York, NY.

**Keywords:** Meta-research, research on research, indexing

## Abstract

Meta-research is a bourgeoning field studying topics with significant relevance to health sciences librarianship, such as research reproducibility, peer review, and open access. As a discipline that studies research itself and the practices of researchers, meta-research spans disciplines and encompasses a broad spectrum of topics and methods. The breadth of meta-research presents a significant challenge for identifying published meta-research studies. Introducing a subject heading for meta-research in the controlled vocabularies of literature databases has the potential to increase the visibility of meta-research, further advance the field, and expand its impact on research practices. Given the relatively recent designation of meta-research as a field and its expanding use as a term, now is the time to develop appropriate indexing vocabulary. We seek to call attention to the value of meta-research for health sciences librarianship, describe the challenges of identifying meta-research literature with currently available key terms, and highlight the need to establish controlled vocabulary specific to meta-research.

## INTRODUCTION

While the term may be unfamiliar, meta-research, or ‘research on research,’ is already having an effect on how research is being performed, evaluated, and disseminated [1-3]. However, in spite of its far-reaching impact, the visibility and accessibility of meta-research as a field has been limited by the lack of a consistent and standardized way of categorizing meta-research in literature databases, such as the lack of a MeSH (Medical Subject Heading) term for searching for “meta-research” in PubMed.

We describe here the challenge of identifying meta-research literature with currently available terms. We then advocate that scholarly citation databases recognize the field of meta-research and establish indexing terms for meta-research that can make it easier to locate meta-research studies. We hope to call attention to the value of meta-research in library and information science, particularly health sciences librarianship.

## WHAT IS META-RESEARCH AND WHY DOES IT MATTER FOR LIBRARIANS?

Meta-research is a relatively newly defined discipline designed to study research itself and its practices. To promote robust science, meta-research uses an interdisciplinary approach to examine research practices with the same scientific rigor given to other areas of scientific inquiry [4]. The objective of this approach is to understand and improve how research is performed, communicated, verified, evaluated, incentivized, and supported. By examining research practices, meta-research can help disseminate efficient and effective research policies and identify and abandon wasteful ones [5]. The use of meta-research is far reaching with topics explored in meta-research covering all aspects of the scientific process, including, but not limited to, publication and peer review models, scientific education, funding, and academic reward systems. Indeed, the products of meta-research are experienced every time an open access paper is read, [1] open peer review is implemented, [2] or a manuscript data-availability statement is written [3].

In the age of paper mills and predatory journals, meta-research provides a framework to examine journal practices and develop solutions to many issues being faced within health sciences librarianship [6, 7]. Much of the work being done within health sciences librarianship is important meta-research that helps shape how research publications are catalogued, evaluated, and managed. In fact, a fair amount of research being published in journals like the *Journal of the Medical Library Association* are examples of meta-research, even if they are not labeled as such. Examples of meta-research scholarship published in the *Journal of the Medical Library Association* include: bibliometric research exploring the presence of predatory journal publications in published literature reviews [7] and the creation of research performance metrics; [8] discussion around the implications of authorship order; [9] exploration of the challenges to research collaborations [10] and the roles played by librarians in systematic reviews; [11] study of the training needs and motivations of researchers [12] and librarians; [13] examination of the weaknesses in current publishing practices; [14] and the development [15] and assessment of search strategies [13].

As the topics explored within meta-research are broad, to better categorize meta-research efforts, it has been proposed that the discipline be divided into the following themes: methods (how research is performed, e.g., study design, analytic approaches, research ethics), reporting (how research is communicated, e.g., reporting standards, information sharing, conflict of interest reporting), replicability and reproducibility (how research is verified or replicated, e.g., methods of data sharing, efforts to reproduce previous studies), evaluation (how research is evaluated, e.g., peer review, funding criteria, research impact), incentives (how research is rewarded or supported, e.g., promotion criteria, developing research capacity), and organization (how research is organized or categorized, e.g., research categorization, interactions between disciplines) [16]. The questions found in meta-research are often answered with many of the same methods used to address other scientific inquiries, whether it be through experimentation, observation, or literature synthesis.

## CHALLENGES TO FINDING META-RESEARCH

Given the broad scope of meta-research,[16] it can be challenging to retroactively define a unifying set of key terms to identify meta-research. Researchers have been performing meta-research for decades on a number of topics and in a variety of disciplines, however, few studies have explicitly self-identified as meta-research or used terms such as “meta-research,” “meta-science,” or “research on research” at any point within their publications or defined keywords. Therefore, to identify the literature existing within meta-research, alternate strategies are needed. Given the nature of the discipline, identifying meta-research is likely to be an insurmountable challenge without establishing an appropriate indexing term, or using methods like machine learning or natural language processing.

While all centered around the subject of research practices, the sheer breadth of meta-research, which is performed across disciplines, topics, and methods, makes any currently existing search term or combination of terms insufficient to envelop the whole of meta-research. This is particularly evident with the many topics explored and methods used in meta-research overlap with common terms and phrases used in most other research fields. Some subsets of meta-research themes do lend themselves more readily to easily distinguishable key terms, such as “research ethics,” “research reproducibility,” or “peer review,” but these topics are more often the exception than the rule. Many meta-research topics have less straight forward keywords, with many of the words used throughout the discussion, methods, and defined key terms overlapping with those used in other topics and/or are commonly used words, in particular the word “research” itself.

One such example of a meta-research topic without easily identified search terms includes research falling within the theme of incentives (i.e., how research is rewarded or supported) [16]. In this research, the subjects are often researchers themselves. Insights from the body of literature with researchers as the study subjects can illuminate how to better support researchers, enhance the development of research capacity, and advance the methods used to engage and study this population. However, it can be a challenge to assess and synthesize the breadth of the meta-research literature produced. For instance, one term for the population studied, “researcher,” is used in tens of millions of published articles within the PubMed database, and therefore a less useful term for identifying a study population than the term “smoker,” for example, which is found in less than one-fourteenth the number of articles. The use of numerous synonyms for researchers, such as “study team,” “staff,” or “librarian” further compound the issue. The MeSH term “Research Personnel” does exist, which may be presumed to capture this literature, however, this term has a broad and vague definition that identifies some, but not all literature with researchers as the subject, and also captures a significant amount of literature if research personnel are mentioned as “doing” or “finding” something but not necessarily the subject of a meta-research study on research practices.

Many other themes within meta-research face a similar challenge to define terms, or combinations thereof, that could be used to accurately, with both sensitivity and specificity, identify appropriate meta-research studies in a literature review. While searches can be developed with existing terms, any attempt to compile the body of meta-research literature is likely to yield an unwieldy collection containing enormous numbers of irrelevant literature or a significantly limited set missing a large portion of the existing meta-research literature. The concern over a broad and poorly delimitated term-set for meta-research could be ameliorated with the development of a controlled vocabulary to index and identify meta-research.

## THE NEED FOR A SPECIFIC META-RESEARCH MESH TERM

Meta-research could strongly benefit from the establishment of its own indexing terms to increase the field's visibility and propel its research forward, as it is currently difficult to locate meta-research within citation databases which lack controlled vocabulary for meta-research [13, 17-18]. As a discipline designed to study research itself and its practices, meta-research is not only multidisciplinary, but also comparably new as a distinct research field [4, 5]. Having been coined less than a decade ago, [19] meta-research is generally lacking widespread recognition or a united set of terminology. Studies that would now be labeled meta-research have been produced for decades on a number of topics and in a variety of disciplines, but rarely under the moniker of “meta-research.” This makes finding meta-research very challenging. To add to the confusion, the term meta-research is also frequently used to refer to meta-analysis, which is a statistical analysis used to combine the data from independent studies on the same subject and is distinct from meta-research the discipline. For these reasons, meta-research is a prime example of where unification of a topic within an index term can make relevant research discoverable across disciplines regardless of the mode du jour or favored terms in a particular research group. Without the use of indexing terms, search results would be limited to specific keywords or phrases used in the text, which could lead to irrelevant or incomplete results due to inconsistences in use of terminology as well as differences such as alternate spellings in different geographic areas.

There is currently no controlled vocabulary for meta-research available in any of the major health literature databases (e.g., MEDLINE and Embase) nor do they exist in databases covering other disciplines. While there are MeSH terms currently available that touch on different aspects and themes of meta-research, the available MeSH terms have significant limitations as there is no term that unifies all of meta-research, nor are all meta-research themes addressed. Furthermore, the terms are, in general, broadly defined, thus capturing unrelated literature. Listed below are some of the existing MeSH terms that describe aspects of meta-research, as well as meta-research concepts which lack specific MeSH terms and are thus difficult to search due to shared vocabulary used broadly beyond meta-research.

### Existing MeSH Terms Related to Meta-Research

- Research
- Research Personnel
- Bibliometrics
- Reproducibility of Results
- Meta-Analysis as Topic
- Open Access Publishing
- Peer Review

Meta-Research Concepts Not Easily Searchable and Lacking MeSH Terms

- Meta-Research
- Research Teams
- Research Impact
- Research Registration/Preregistration
- Research Incentives
- Research Funding
- Open Science

The various meta-research being performed has potentially vast implications for how institutions and the research community support, perform, and present research. However, the scope of knowledge gained through meta-research remains relatively unknown and inaccessible due to the limitations of available key terms. The homogeneity that exists within meta-research (the shared focus on researching research practices) and thus the potential impact of solidifying meta-research as a discipline make creating a meta-research MeSH term both useful and important. Furthermore, the establishment of a meta-research MeSH term may encourage the development of a similar term in the controlled vocabularies of other databases in other disciplines. A possible format for the MeSH terms could be a subject heading of “meta-research” with a subheading for each meta-research theme.

The availability of defined index terms has the potential to shape the meta-research discipline and the lack of meta-research indexing terms has likely limited the visibility and impact of the field. A MeSH term unifying the whole of meta-research can help crystalize what meta-research the discipline is and bring attention to the work being done in the field. This can have downstream effects impacting the trajectory of the discipline such as availability of funding and acceptance of meta-research into higher-impact journals. Therefore, defining controlled vocabulary specific to meta-research may improve the production and dissemination of meta-research.

The NLM includes a process to consider user suggestions for MeSH terms, which are reviewed by NLM staff for need, usefulness, and understandability[20]. In other areas NLM has been responsive, creating new MeSH terms when gaps have been identified [21, 22]. Similarly, as MEDLINE has expanded to incorporate an increasing number of indexed public health, life and physical sciences journals, MeSH has expanded its scope accordingly to incorporate the new controlled vocabularies associated with these fields [23]. As meta-research is already being published in MEDLINE-indexed journals, it is worth considering the addition of MeSH terms specific to the field.

While meta-research's interdisciplinary nature, broad scope, and difficult-to-pinpoint terminology make the complete understanding of the meta-research literature challenging, it is evident that meta-research is being published across many MEDLINE-indexed journals. As a discipline, however, meta-research remains left out of the benefits derived from a defined MeSH term. For this reason, an application will be pursued with the NLM to establish a MeSH term for “meta-research.” This is one step in addressing the ongoing need to raise awareness of meta-research and how it is already impacting how research is being done. To support these efforts, librarians can write an open letter to the NLM supporting the creation of a MeSH term for meta-research. Furthermore, more broadly, librarians can recognize how they are involved in and contributing to meta-research and label their work as meta-research.

While a meta-research MeSH term would make it easier to find meta-research published in the health sciences (and may spur databases in other fields to add a term for “meta-research” in their controlled vocabularies), the development and use of a meta-research MeSH term has its limitations and would not be a complete panacea to finding all meta-research literature. MeSH terms are only applied prospectively, so the literature that already exists will not be included under the index term. Similarly, because the themes of meta-research are so broad, future research may be missed when indexing depending on the definitional decisions made. Therefore, alternate methods for searching the literature are also worth entertaining. Methods such as machine learning and natural language processing may serve as useful strategies in addition to the categorization of meta-research under a MeSH term.

## CONCLUSION

Meta-research is a bourgeoning field that spans disciplines and encompasses a broad spectrum of topics and methods. Its breadth makes defining a set of its key terms a significant challenge. Defining controlled vocabulary for identifying meta-research has the potential to further advance the field and expand its impact on research practices. Given the relatively recent designation of meta-research as a field and its expanding use, now is the time to develop appropriate indexing terms and expand the impact of contributions to meta-research from professions such as health sciences librarianship.
